# Porcine hepatocytes culture on biofunctionalized 3D inverted colloidal crystal scaffolds as an *in vitro* model for predicting drug hepatotoxicity

**DOI:** 10.1039/c9ra03225h

**Published:** 2019-06-07

**Authors:** Lingyan Wu, Gaia Ferracci, Yan Wang, Teng Fei Fan, Nam-Joon Cho, Pierce K. H. Chow

**Affiliations:** Division of Surgical Oncology, National Cancer Centre Singapore 11 Hospital Drive 169610 Singapore pierce.chow.k.h@singhealth.com.sg; Interdisciplinary Graduate School, NTU Institute for Health Technologies, Nanyang Technological University 50 Nanyang Drive 637553 Singapore; School of Materials Science and Engineering, Nanyang Technological University 50 Nanyang Avenue 639798 Singapore njcho@ntu.edu.sg; Centre for Biomimetic Sensor Science, Nanyang Technological University 50 Nanyang Drive 637553 Singapore; School of Chemical and Biomedical Engineering, Nanyang Technological University 62 Nanyang Drive 637459 Singapore; Duke-NUS Medical School 8 College Road 169857 Singapore; Department of Hepatopancreatobiliary and Transplant Surgery, Singapore General Hospital Outram Road 169608 Singapore

## Abstract

As drug-induced hepatotoxicity represents one of the most common causes of drug failure, three-dimensional (3D) *in vitro* liver platforms represent a fantastic toolbox to predict drug toxicity and thus reduce *in vivo* animal studies and lessen drug attrition rates. The aim of this study is to establish a functional porcine hepatocyte culture using a biofunctionalized 3D inverted colloidal crystal (ICC) hydrogel platform. The performances of non-adhesive bare poly(ethylene glycol)diacrylate (PEGDA) ICCs and PEGDA ICCs coated with either collagen type I or fibronectin have been investigated. Porcine hepatocytes viability, morphology, hepatic-specific functions and patterns of gene expression have been evaluated over a period of two weeks in culture to test diclofenac, a well-known hepatotoxic drug. Interestingly, cells in the fibronectin-functionalized scaffold exhibit different aggregation patterns and maintain better liver-specific function than those in bare ICCs and in collagen functionalized scaffold. We concluded that the 3D cell culture environment and the presence of extracellular matrix (ECM) proteins, especially fibronectin, facilitate hepatocyte viability and maintenance of the liver-specific phenotype *in vitro*, and enable us to predict hepatotoxicity.

## Abstract 2

As drug-induced hepatotoxicity represents one of the most common causes of drug failure, three-dimensional *in vitro* liver platforms represent a fantastic toolbox to predict drug toxicity and reduce *in vivo* studies and drug attrition rates.

## Graphical abstract

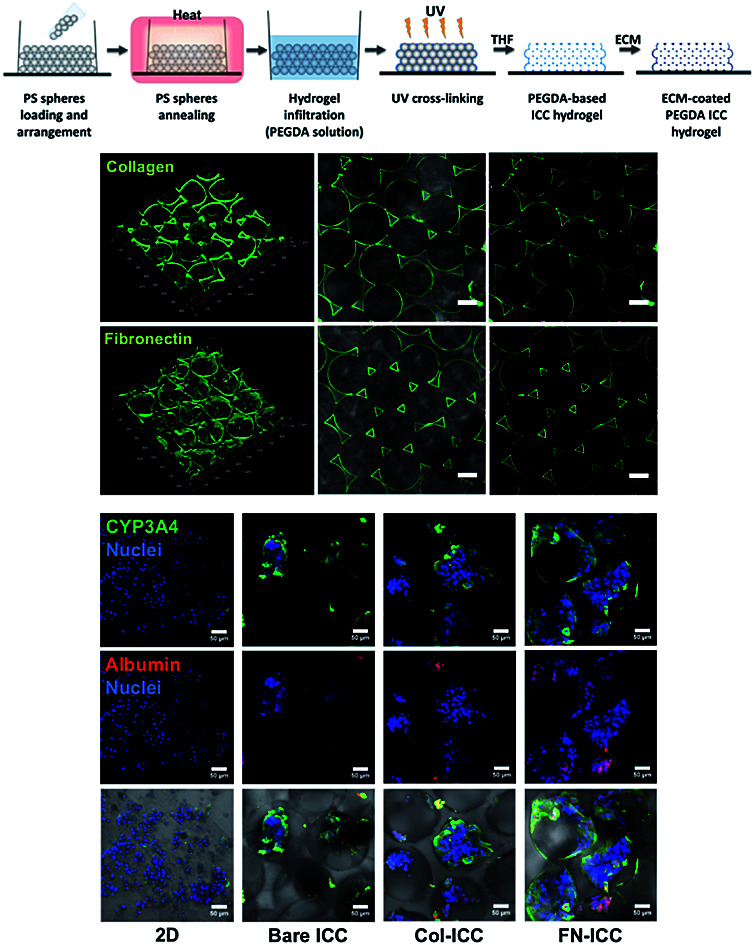

## 1. Introduction

Drug-induced liver injuries (DILIs) still is a major concern for the pharmaceutical industry and regulatory authorities as well as for patients and clinicians.^1,2^ Indeed, drug-induced hepatotoxicity is among the most common reasons for drug failure at the clinical and post-marketing phases.^1,3,4^ It has been reported that, among the medicinal products withdrawn between 1953 and 2003 due to adverse drug reactions, around 60% were removed from the market because of toxicity issues, 30% of which related to hepatotoxicity.^5^ Excluded from the count were traditional Chinese medicinal products and dietary supplements, which, in recent years, have been increasingly reported to be associated with liver injury, with varying occurrence across countries.^6–8^ DILI is also among the main causes of acute liver failure in the USA and Europe, a life-threatening condition whose ultimate therapeutic option is liver transplantation.^1,3,9^

In order to minimize the risk for patients and volunteers, the efficacy and safety of new potential drug candidates are generally evaluated through preclinical *in vitro* assays and *in vivo* studies. *In vivo* studies usually involve single or multiple administrations of the pharmaceutical compounds to two mammalian species (one of which should be non-rodent) and observation of the drug effects over a period of several weeks or months, depending on the proposed usage in humans. However, these studies are highly costly and time-consuming, as they require a large number of animals over extended periods of time, and are not always predictive of human toxicity.^10,11^

One of the main reasons why drug-induced hepatotoxicity couldn't be accurately predicted during preclinical animal studies resides in the inter-species variations in the composition, expression, and activity of the hepatic drug metabolizing enzymes. Indeed, no single animal species completely resemble humans in terms of drug metabolism, although some species possess similarities with regard to specific CYP enzymes activity.^12^

Therefore, *in vitro* liver platforms for drug testing based on hepatocytes from different animal species might be a potent tool for high throughput screening to reduce *in vivo* animal studies during drug development and ultimately lessen drug attrition rates and costs.

Many studies demonstrated that hepatocytes cultured on conventional two-dimensional (2D) substrates rapidly dedifferentiate, with loss of viability and phenotype within one week of culture.^13–16^ Indeed, cellular functions are strongly correlated with the architectural arrangement and characteristics of the *in vivo* microenvironments, such as three-dimensionality (3D), cell–cells interactions and cell–extracellular matrix (ECM) interactions, are lost in 2D monolayer cultures. In order to retain hepatocytes viability and functionality over longer periods of time a number of different strategies, mimicking different aspects of the complex *in vivo* liver microenvironment, have been proposed.^17–20^ Though coating of 2D substrata with ECM proteins helps in maintaining liver-specific functions to a greater extent than conventional tissue culture plates, 3D architecture plays a prominent role in improving such functions,^21–23^ highlighting the importance of recapitulating more than one of the *in vivo* aspects when developing artificial liver platforms.

Among the various 3D systems proposed, inverted colloidal crystal (ICC) scaffolds seem to be a promising platform to mimic the hepatic lobular organization towards functional liver tissue. Indeed, ICCs are obtained as the inverse replica of a 3D array of hexagonally-packed monodispersed microspheres and, as such, these scaffolds possess interconnected and uniform pores and, consequently, reproducible and tunable physical properties and efficient diffusion of nutrients, waste products and oxygen.^24^ Kotov and colleagues showed that ICCs made of non-fouling materials, such as poly(acrylamide) (PAAm), could be used to produce a large number of spheroids of precise diameter. HepG2 cells cultured in PAAm ICCs aggregated into spheroids within the ICC cavities, and these hepatospheres showed an inner network of channels similar to the *in vivo* bile canaliculi and reduced toxicity to gold nanoparticles compared to 2D culture.^25,26^

Our group has previously shown that functionalization of non-adhesive poly(ethylene glycol)diacrylate (PEGDA) ICC hydrogels with ECM proteins promoted the formation of cell-sheet like aggregates lining the wall of the ICC cavities. Huh-7.5 cells in this configuration shown improved viability and hepatic-specific functionality compared to 2D cultures and 3D spheroids obtained with non-functionalized PEGDA ICCs hydrogels, highlighting the importance of a 3D *in vivo* like structure, with cell–cell as well as cell–ECM interactions, to obtain functional liver tissue *in vitro*.^27,28^ Our previous studies further showed that functionalization with either collagen type I or fibronectin differentially regulated liver cell phenotype.^29^

The aim of this study was to establish a functional porcine hepatocyte culture that can be used to predict drug toxicity using biofunctionalized 3D ICC hydrogels. In order to test the drug toxicity, cultured hepatocytes were assessed after single or repeated exposure to diclofenac, a non-steroidal anti-inflammatory drug (NSAID) widely prescribed worldwide for the treatment of rheumatic and pain and associated with rare but severe cases of liver toxicity.^30,31^ Porcine hepatocytes were chosen for their biotransformation ability similar to human hepatocytes. We investigated the performances of non-adhesive bare PEGDA ICCs, and PEGDA ICCs coated with either collagen type I or fibronectin, the most abundant proteins in the liver parenchyma.^32^ Hepatocytes viability and functionality were evaluated over a period of 2 weeks in culture and after repeated exposures to the hepatotoxic drug.

## 2. Experimental section

### 2.1 Poly(ethylene glycol)-diacrylate (PEGDA) synthesis

PEGDA was synthesized following previously reported methods.^33,34^ Shortly, diol-terminated PEG (Mw = 4600; Sigma-Aldrich) was reacted (overnight, room temperature) with acryloyl chloride (Merck) and triethylamine base catalyst (2.5 molar excess; T0886; Sigma-Aldrich) in tetrahydrofuran (THF; Sigma-Aldrich). PEGDA was purified by filtration, extracted in dichloromethane and precipitated in diethyl ether.

### 2.2 PEGDA-based inverted colloidal crystal (ICC) hydrogels fabrication

**Fig. 1:**
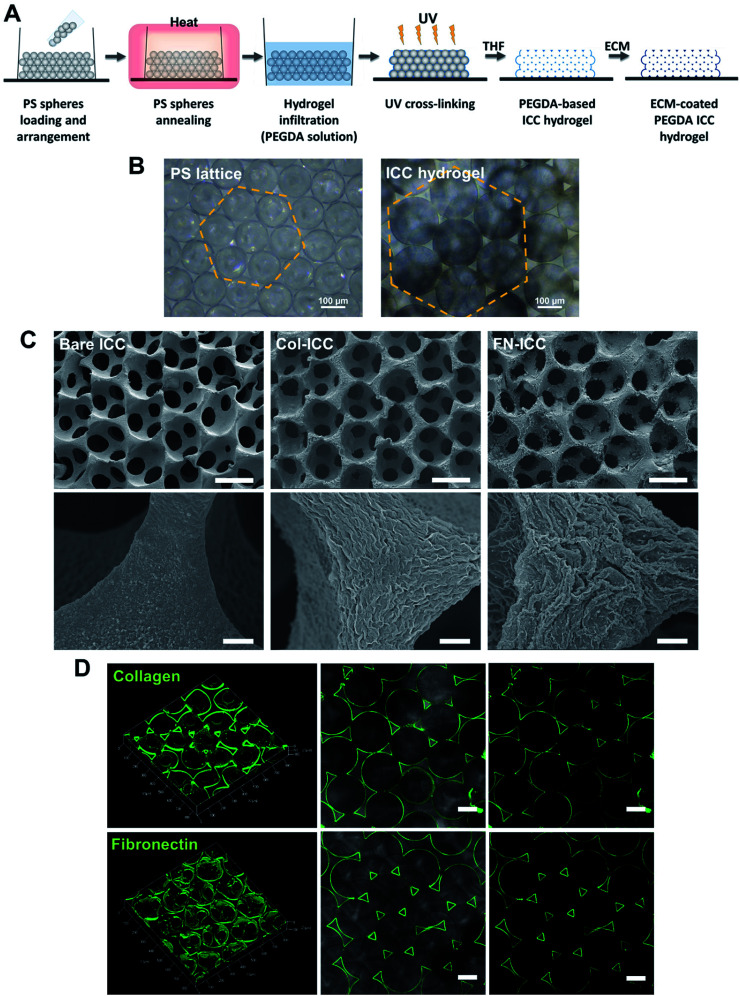
PEGDA-based ICC hydrogels. (A) Schematic illustration of the fabrication process. ICC scaffolds are prepared as the inverse replica of an ordered array of self-assembled monodisperse microspheres. PS, polystyrene; PEGDA, poly(ethylene glycol)diacrylate; UV, ultraviolet; THF, tetrahydrofuran; ICC, inverted colloidal crystal; ECM, extracellular matrix. (B) Optical microscopy images of annealed PS beads self-assembled in a hexagonal manner (left) and PEGDA ICC hydrogels with pores maintaining the same geometrical arrangement (right). Scale bars = 100 μm. (C) SEM images of bare PEGDA ICCs (Bare ICCs; left), collagen-coated PEGDA ICCs (Col-ICCs; middle) and fibronectin-coated PEGDA ICCs (FN-ICCs; right) at different magnification, showing the highly regular and porous scaffold structure, with interconnected pores (top; scale bar = 100 μm), and the surface differences of the pore walls in the presence of ECM coating (bottom; scale bar = 5 μm). (D) Confocal images of the PEGDA ICC scaffolds functionalized with 200 μg mL^−1^ collagen type I (top) or fibronectin (bottom). ICCs were stained with specific anti-collagen I or anti-fibronectin antibody and green-fluorescent Alexa Fluor 488. 3D reconstruction using ZEN software (left) and 2D images (middle and right). A thin uniform coating is observed in both cases. Scale bar = 100 μm.

PEGDA-based ICC hydrogels were fabricated following a protocol previously described.^35^ PS spheres (139 ± 2.9 μm; Duke Scientific) were washed in ethanol (75% v/v), ultra sonicated (1 minute, 3–4 times), loaded into molds obtained by gluing polypropylene tubes (i.d. = 6 mm) onto glass slides and shaken using a rocking platform (overnight; room temperature; VWR). If need be, the arrangement of spheres in a hexagonal fashion was obtained by additional manual and mechanical shaking (2 days; room temperature). The PS lattice template was obtained by annealing the microspheres in a hoven (136 °C, 6 h). PS lattices were infiltrated with the hydrogel precursor solution (300 μL per PS lattice) by centrifugation (845 × *g* for 5 min), blotted dry and irradiated with UV light (UV chamber, 365 nm, 6 min). The hydrogel precursor solution was obtained by adding the photoinitiator 2-hydroxy-4′-(2-hydroxyethoxy)-2-methylpropiophenone (Irgacure 2959, 0.1% w/v), previously dissolved in ethanol (20% w/v), to a PEGDA solution (50% w/v in deionized water). For ECM-coated ICC scaffolds, acryloyl-PEG-*N*-hydroxysuccinimide (PEG-NHS; 10% w/v; Laysan Bio) was also added to the PEGDA solution. Photopolymerized hydrogels crystal lattices were then soaked in THF and shaken on an orbital shaker (300 rpm, 24 h), in order to completely dissolve the PS spheres. After, ICCs were disinfected in ethanol (1 h), washed with PBS (30 min, three times) and sterilized under UV light (1 h each side). The surface of PEGDA-PEG-NHS ICCs only was further coated with ECM proteins, exploiting the crosslinking reaction between the NHS-ester and the free amino groups on the collagen and fibronectin. PEGDA-PEG-NHS ICCs were soaked in collagen type I (collagen type I from rat tail; Sigma; Sigma-Aldrich) or fibronectin (Corning) solution (200 μg mL^−1^ in PBS) on an orbital shaker (400 rpm, 30 min, room temperature), stored at 4 °C overnight and washed with PBS (three times). Fig. 1A depicts the ICC fabrication process. All the ICC were stored in PBS (4 °C) until used.

### 2.3 ICCs characterization

ICCs morphology was observed with Scanning Electron Microscopy (SEM). For SEM analysis, hydrogels were fixed with paraformaldehyde (PFA; 4%, 10 min; Alfa Aesar), sequentially dehydrated with increasing concentrations of ethanol (25%, 50%, 75%, 95% and 100%; 30 min each), freezed (−80 °C, overnight) and freeze-dried (48 h; FreeZone 4.5 litre freeze-dryer; Labconco). Samples (top surface) were coated with a platinum (Pt) film (10 nm) in a JFC-1600 sputter coater (JEOL) (20 mA, 60 s) and images were acquired with a FESEM 7600F (JEOL; 5 kV, different magnifications).

Collagen- or fibronectin-coating of ICC hydrogels was evaluated by immunofluorescence. Briefly, Col-ICCs and FN-ICCs were fixed with PFA (4%, 10 min), washed with PBS and incubated (4 °C, overnight) with a mouse anti-collagen I primary antibody (1 : 100; Abcam) or a rabbit anti-fibronectin primary antibody (1 : 100; Abcam), respectively, in a bovine serum albumin (BSA; Sigma-Aldrich) solution (3% w/v in PBS). ICCs were then washed with PBS and incubated with a goat anti-mouse (Life Technologies) or a goat anti-rabbit (Life Technologies) secondary antibody conjugated with Alexa Fluor 488 (1 : 100; 2 h). Fluorescent images were acquired with an LSM710 confocal microscope (Carl Zeiss) equipped with an Axio Observer Z1 inverted microscope (Carl Zeiss). Reconstructed 3D images were obtained using ZEN software (Zeiss).

### 2.4 Isolation of pig hepatocytes

**Fig. 2:**
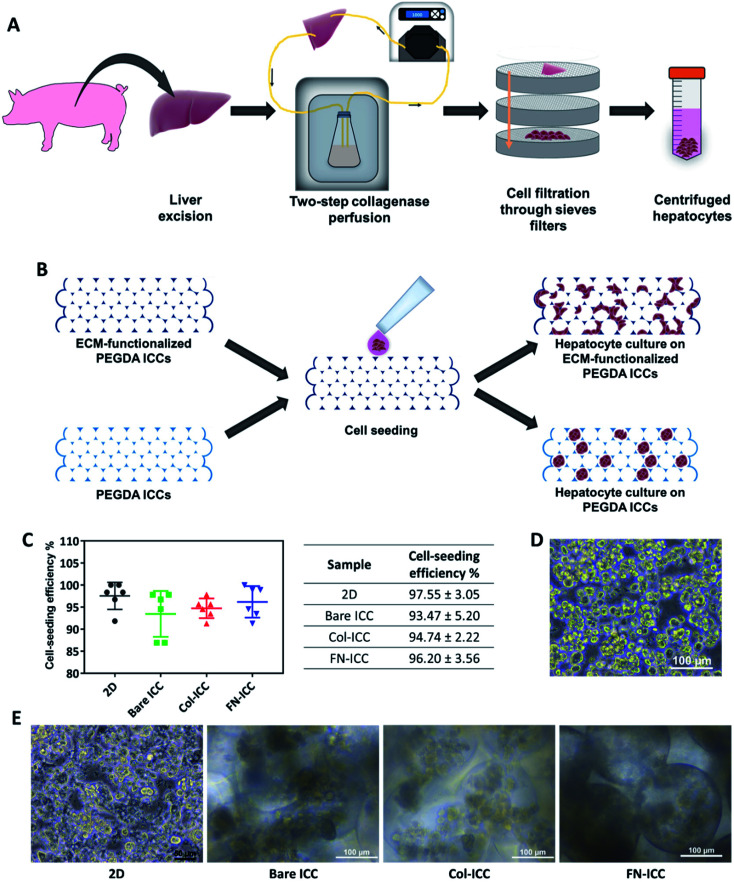
Cell seeding on PEGDA-based ICC hydrogels. Half million hepatocytes were seeded on either 2D multiwell plates or ICC hydrogels. (A) Schematic illustration of the porcine hepatocyte isolation process. Hepatocytes were isolated from pig liver by a two-step collagenase perfusion method. (B) Schematic illustration of cell seeding and hepatocyte culture on non-adhesive PEGDA ICCs and PEGDA ICCs functionalized with ECM proteins (collagen type I and fibronectin). (C) Cell seeding efficiency was quantified 24 h after seeding by calculating the number of cells not attached to the 2D or 3D substrata. Data are reported as mean ± SD (*n* = 6). (D) Optical microscopy image of freshly isolated hepatocytes after the two-step collagenase perfusion. Scale bar = 100 μm. (E) Optical microscopy images of the porcine hepatocytes 1 day after seeding on 2D wells (scale bar = 50 μm) or ICC hydrogels (scale bar = 100 μm).

All animal procedures were performed in accordance with the Guidelines for Care and Use of Laboratory Animals of the Institutional Animal Care and Use Committee at SingHealth Research Singapore and approved by the Animal Ethics Committee of SingHealth Research Singapore. Pigs (16–20 weeks old, 15–20 kg) were sedated with an intramuscular injection of ketamine/xylazine (15 mg kg^−1^/2.2 mg kg^−1^) and, after endo-tracheal intubation, anesthesia was maintained with inhalational isoflurane (1–2%). Heparin sodium (12500 U) was injected into the lateral auricular vein and open laparotomy was performed. Hepatocytes were isolated from pig liver by a two-step collagenase perfusion method.^36–38^ The liver was immediately excised and the left lateral segment was removed and put in a stainless-steel container. The left hepatic artery and the left bile duct were ligated, while the left portal vein was cannulated and connected to a peristaltic pump (Cole-Parmer) in order to be used as the inflow during the perfusion steps. The left hepatic vein, instead, was used as the outflow. To wash out all the blood, the lobe was perfused with warm perfusion buffer I (450 mL, 30 mL min^−1^) and, after, washed with warm perfusion buffer II (250 mL, 30 mL min^−1^). Perfusion buffer I consisted of Hank's Balanced Salt Solution (HBSS; Gibco, Life Technologies) supplemented with BSA (0.2 mg mL^−1^; Sigma-Aldrich), EGTA (0.5 mM; Sigma-Aldrich) and ascorbic acid (50 mg L^−1^; Sigma-Aldrich). Perfusion buffer II consisted of Dulbecco's Modified Eagle's Medium (DMEM; Gibco, Life Technologies) supplemented with BSA (0.2 mg mL^−1^; Sigma-Aldrich). Successively, the lobe was perfused with warm digestion buffer (900 mL, 30 mL min^−1^, 30 min). Digestion buffer consisted of DMEM supplemented with BSA (0.2 mg mL^−1^; Sigma-Aldrich), collagenase type IV (0.5 mg mL^−1^; Sigma-Aldrich) and DNAse (0.3 mg mL^−1^; Sigma-Aldrich). The lobe was then transferred to stainless steel sieves. The remaining collective tissue was removed and the liver cells were released by disruption of the capsule and subsequent raking of the liver stroma with a Teflon spatula. Cells were filtered through a series of sieves filters of decreasing mesh size (850 mm, 500 mm and 100 mm; Cole-Parmer), in order to remove debris. Filtered cells were then washed by low speed centrifugation (75 g, 5 min) and the resulting pellet, rich with polyploid liver cells, was resuspended in ice cold plating media (50 mL). Plating media was prepared by supplementing DMEM/F12 (Gibco, Life Technologies) with foetal bovine serum (FBS, 5%; HyClone, GE Healthcare Life Science), insulin (10 μg mL^−1^; from bovine pancreas, I0516; Sigma-Aldrich), dexamethasone (1 μM; Sigma-Aldrich) and penicillin and streptomycin (100 U mL^−1^ penicillin, 100 μg mL^−1^ streptomycin; Gibco, Life Technologies). The cell suspension was kept on ice during counting. The cell suspension was further washed by centrifugation (75 g, 5 min), to remove hematopoietic cells in the supernatant, filtered using a nylon mesh (70 μm), to remove clusters, and washed again by centrifugation (75 g, 5 min) before proceeding to count cells. Fig. 2A depicts the hepatocyte isolation process.

### 2.5 Cell seeding and cell culture

Immediately before cell seeding, ICC hydrogels were pre-conditioned with media (1 mL, 30 min) and successively let dry (1 h).

Isolated pig hepatocytes, stained with trypan blue (Sigma-Aldrich) in order to assess viable cells, were counted using an automated cell counter (Bio-Rad), by setting a cell size range of 16–28 μm. Centrifuged cells were resuspended in plating media (20 × 10^6^ cells per mL) and the cell suspension (25 μL, 0.5 × 10^6^ viable hepatocytes) was pipetted on top of each ICC. Hydrogels were maintained in a humidified atmosphere (37 °C, 5% CO_2_, 4 h) until cells attached to the scaffolds. After, plating media (1 mL) was added in each well containing ICC. As control, hepatocytes were seeded on 24-well tissue culture plates. After 24 h, cell-seeded ICCs were transferred to new 24-well plates and in all the samples (both 2D and 3D) the plating media was replaced with culture media. Culture media was prepared by supplementing DMEM/F12 with BSA (0.2 mg mL^−1^; Sigma-Aldrich), insulin, transferrin, selenium and ethanolamine (10 μg mL^−1^, 5.5 μg mL^−1^, 6.7 ng mL^−1^ and 2 ng mL^−1^, respectively; Gibco, Life Technologies), l-glutamine (2 mM; Gibco, Life Technologies), dexamethasone (1 μM), linoleic acid (1.5 μg mL^−1^; Sigma-Aldrich) and penicillin (100 U mL^−1^), streptomycin (100 μg mL^−1^) and amphotericin B (0.25 μg mL^−1^). Cells were cultured in a humidified atmosphere (37 °C, 5% CO_2_) up to 14 days. Culture media was changed every 2 days.

### 2.6 Cell-seeding efficiency and cell viability

Cell-seeding efficiency was evaluated counting the number of seeded cells unattached to the scaffolds. 24 h after seeding, ICCs were transferred to new 24-well plates and cells remaining in the former 24-well plates (both cells in the plating media and cells attached to the bottom of the wells) were counted using an automatic cell counter. A cell size range of 16–28 μm was used to take only hepatocytes into account. Trypan blue was utilized to check the viability of unattached hepatocytes.

Cell viability was measured using the Cell Counting Kit-8 (CCK-8; Dojindo Molecular Technologies) assay and qualitatively evaluated using the Live/Dead Cell Viability/Cytotoxicity kit (Life Technologies) according to manufacturers' protocol.

For CCK-8 measurements, CCK solution (500 μL, 1 : 10 CCK : culture media) was added to each well (37 °C, 2 h) and the absorbance at 450 nm was measured using an Infinite 200 PRO microplate reader (Tecan).

For Live/Dead analysis, calcein-acetomethoxy (calcein-AM, 6 μM) and Ethidium homodimer-1 (EthD-1, 4 μM) in culture media were added to each well (500 μL, 37 °C, 1 h). Then, samples were washed with PBS and live cells, stained green, and dead cells, stained red, were visualized using an LSM710 confocal microscope equipped with an Axio Observer Z1 inverted microscope. Z-stack images were processed and sum slices projection images were generated using ImageJ software.

### 2.7 Cell functionality assays

To evaluate hepatocytes specific functions, albumin and urea secreted in the media during a 24 h period were measured using a pig albumin enzyme-linked immunosorbent assay (ELISA) kit (Abcam) and a urea assay kit (Abcam), respectively. Assays were conducted according to manufacturers' instructions. Albumin and urea concentration values were normalized against the nutrient medium volume and the number of seeded cells.

### 2.8 Immunofluorescence staining

To assess the spatial distribution of hepatic cell-specific functions within the ICC scaffolds, immunofluorescence staining of albumin and CYP3A4 was performed. At selected time points, ICCs or 2D culture were washed with PBS (twice), fixed with cold PFA (4%, 30 min, room temperature) and washed again with PBS (5 min, twice). ICCs and 2D culture were then permeabilized and blocked (45 min, room temperature) with TritonX-100 (0.1% w/v; ThermoScientific) in BSA solution (5% w/v in PBS, 100 μL). Samples were successively incubated (4 °C, overnight) with either goat anti-albumin primary antibody (1 : 100; Abcam) or rabbit anti-CYP3A4 primary antibody (1 : 200; Merck) in BSA (2% w/v in PBS, 80 μL). Afterward, ICCs and 2D culture were washed with PBS (5 min, four times), to remove unbound primary antibody, and incubated (45 min, room temperature, protected from light) with either anti-goat secondary antibody conjugated with Alexa Fluor 594 (1 : 500; Invitrogen) or anti-rabbit secondary antibody conjugated with Alexa Fluor 488 (1 : 500; Invitrogen) in BSA (2% w/v in PBS; 80 μL). In order to simultaneously stain the nuclei, Hoechst 33342 (H33342, 1 : 500; Invitrogen) was also added to the secondary antibody solution. Samples were washed with PBS (5 min, twice) before being imaged using an LSM710 confocal microscope equipped with an Axio Observer Z1 inverted microscope. Z-stack images were processed and sum slices projection images were generated using ImageJ software.

### 2.9 Reverse transcription-quantitative real time polymerase chain reaction (RT-qPCR)

The expression of liver-specific functional and regulative genes was determined by RT-qPCR. Total RNA in each sample (2D wells or ICCs at day 14) was isolated using TRIzol reagent (Invitrogen) following manufacturer's instructions and RNA concentration was then measured using a NanoDrop 2000c spectrophotometer (ThermoFisher Scientific). RNA was reverse-transcribed to complementary DNA (cDNA) using iScript Reverse Transcription Supermix (Bio-Rad), according to manufacturer's instructions. Reverse transcription was conducted by incubation in a thermal cycler (Bio-Rad). cDNAs were amplified using specific primers and SYBR select Master Mix for CFX (Life Technologies), following manufacturer's instructions, in a CFX Connect Real-time PCR detection system (Bio-Rad). Data were analyzed utilizing the 2^−ΔΔ*C*_T_^ method. The expression level of each gene was normalized to the expression level of the housekeeping gene glyceraldehyde-3-phosphate dehydrogenase (GAPDH) and presented as fold change relative to the 2D group. Primers sequences used for cDNA synthesis were selected from the literature and are listed in Table 1.

**Table 1:** Primer sequences used in RT-qPCR: albumin (ALB), α-fetoprotein (AFP), hepatocyte nuclear factor 1-alpha (HNF1α), hepatocyte nuclear factor 4-alpha (HNF4A), cytochrome P450 2C34 (CYP2C34), cytochrome P450 3A29 (CYP3A29), cytochrome P450 1A1 (CYP1A1), glutathione *S*-transferase α1 (GSTA1), glutathione *S*-transferase α2 (GSTA2), glutathione *S*-transferase α4 (GSTA4), glyceraldehyde-3-phosphate dehydrogenase (GAPDH)

Target gene	Forward (5′–3′)	Reverse (5′–3′)	Ref.
ALB	TGTTGCTGATGAGTCAGCTGA	TGGAAGTCAGCGCATAAAGCA	39
AFP	AGATGCCCATAAACCCTGGT	CCAGTAGTCCAGAGAAATCTGCA	39
HNF1α	CTGGCATCTTGCCTCTACTGGAAA	CACGACTCAACCTGGCACCAA	39
HNF4α	ACCAAGCGCTCTATGGTGTTTAAGG	CACAGACACCCGGTTCATTTCTG	39
CYP2C34	CACCAGATGGCGTTTACTT	GTCAATCTCTTTCTGCACTTTG	40
CYP3A29	TTACACTTACCTGCCCTTTG	GCCCTTGCGTGGTTAAT	40
CYP1A1	CAGTACCACAAGAGACACAAG	CCATCGGCAGTGAGAAAC	40
GSTA1	CAGAAGGTGTGGCAGATTT	GTCTTGTCCATGGCTCTTC	40
GSTA2	CAGGGCCATCCTCAATTAC	GCCACCTTGGCATCTTT	40
GSTA4	ACGTGAGGACCGTGTATAA	GACCAACTGAGGAACAAGATAC	40
GAPDH	ACCCAGAAGACTGTGGATGG	CTCAGTGTAGCCCAGGATGC	41

### 2.10 Drug toxicity studies

To evaluate the ability of pig hepatocytes cultured on ICC hydrogels to correctly identify toxic drugs, 2D cultures and cell-seeded ICCs were incubated with diclofenac (200 μM; ≈25*C*_max_; Sigma-Aldrich). *C*_max_ refers to the maximum compound concentration in human blood upon single-dose administration at the recommended therapeutic dose (≈8.023 μM).^42^ To test acute toxicity, 2D cultures and ICCs were incubated with the drug for 24 h, and drug cytotoxicity was evaluated immediately after. To test chronic toxicity, 2D cultures and ICCs were further incubated with the drug, changing culture media every other day (7 drug exposure). Drug cytotoxicity was analyzed by evaluating cell viability, using CCK-8 assay, and hepatic specific functions such as the albumin and urea secreted into the culture media using respectively a pig albumin ELISA kit and a urea assay kit. All the assays were conducted according to manufacturers' instructions. 2D cultures and ICCs incubated with culture media alone were used as a control. A scheme of the experimental timeline for the drug toxicity studies is depicted in Fig. 5A.

### 2.11 Statistical analysis

Data were analyzed using Prism software (GraphPad version 7.0) and quantitative results are presented as mean ± standard deviation (SD) from triplicate samples for each condition. Data shown are from one representative experiment (out of 3). Statistical significant differences between groups were evaluated using one-way or two-way (in drug toxicity studies) analysis of variance (ANOVA) followed by Tukey's multiple comparison test. Statistical significance level was set at *p* < 0.05 and *p* < 0.01.

## 3. Results and discussion

### 3.1 Fabrication and characterization of PEGDA-based ICC hydrogels

PEGDA-based ICC hydrogels were fabricated by the inverse replica of a highly ordered array of monodisperse microspheres, as previously described in detail.^27–29,35,43^ A schematic illustration of the ICC hydrogels fabrication process is given in Fig. 1A. Polystyrene beads, with a mean diameter of 139 ± 2.9 μm and self-assembled in a honeycomb pattern, were used as the sacrificial crystal lattice after undergoing annealing process. ICC scaffolds were obtained after crosslinking the hydrogel precursor solution infiltrated into the voids of the lattice and dissolving the colloidal crystal template (Fig. 1B). The microbeads packed in a hexagonal manner accounted for the highly ordered porous structure of the ICC hydrogels, as well as for the high degree of interconnected pores, as visible in the SEM images (Fig. 1C). Magnification of the hydrogel pore walls shows differences between the surface of bare and functionalized PEGDA ICCs, with fibril-like structures visible on the latter, suggesting the presence of collagen and fibronectin. Coating of the surface of the pore walls with ECM proteins was obtained exploiting the reaction between the *N*-hydroxysuccinimide (NHS) ester conjugated to the poly(ethylene glycol) and the amino groups on the ECM proteins and was verified by confocal microscopy (Fig. 1D).^44^ As observed in the fluorescent images, both collagen and fibronectin formed a thin uniform layer on the hydrogel surface, confirming the coating of the scaffold pore walls. Swelling of the hydrogels in aqueous conditions and shrinking of the scaffolds following dehydration account for the different pores diameter observed in the optical and electronic microscopic images, respectively.

### 3.2 Hepatocyte seeding and viability

Porcine hepatocytes were isolated from pig livers through a two-step collagenase perfusion method (Fig. 2A) with cell viability of 96%, as estimated by trypan blue exclusion. Hepatocytes were isolated mainly as single cells with small aggregates (Fig. 2D). Half million hepatocytes were seeded on either 2D wells or the top surface of ICC scaffolds. As visible in the optical images (Fig. 2E), hepatocytes attached to the 2D substrata and formed an almost confluent layer within 24 h, with some cells maintaining a round shape. In the ICC hydrogels, cells spread over the top surface quite homogeneously and infiltrated into the porous structure, but showed a different adhesive behavior depending on whether or not PEGDA was functionalized with ECM proteins. When hydrogels were not coated with ECM proteins, hepatocytes aggregated together at the center of the pores forming small clusters, with single cells still visible. Conversely, when hydrogels were coated with either collagen or fibronectin, the main components of the liver ECM, hepatocytes adhered to the pore walls, forming aggregates lining the scaffold cavities. Cell-seeding efficiency was calculated after 1 day by counting the cells unattached to the ICC scaffolds or the 2D wells (Fig. 2C). Cell seeding efficiency was slightly higher in 2D substrata (97.55 ± 3.05%) compared to ICCs and slightly higher in ICCs coated with collagen (94.74 ± 2.22%) or fibronectin (96.20 ± 3.56%) than in non-functionalized ICCs (93.47 ± 5.20%), though differences were not statistically significant. The porous structure of ICCs might account for the small differences in cell loading between 2D and 3D substrata, since the highly interconnected and open pores of the ICC allow the release on the bottom of the well of some of the cells seeded on top of the hydrogels, as already reported by Lee and colleagues.^26^ The presence of ECM proteins, instead, might account for the small differences in cell loading between functionalized and bare ICCs, since the presence of adhesion sites on the hydrogel surface might allow the cells to attach to the cavities preventing their release from the bottom of the scaffolds. Fig. 2B depicts the cell seeding process and the hepatocyte culture pattern on the different hydrogels.

**Fig. 3:**
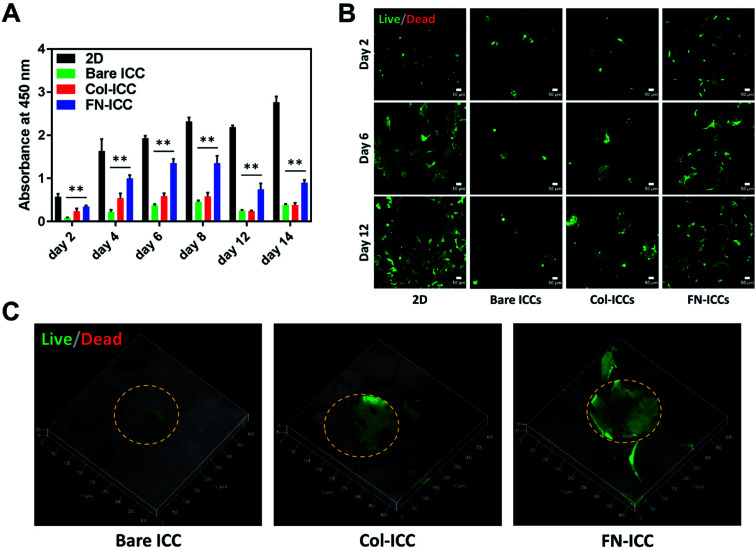
Cell viability in ICC scaffolds. Half million cells were seeded in 2D and 3D ICC scaffolds with or without conjugated Col-I and FN. (A) Cell viability was determined by the CCK-8 colorimetric assay spectrophotometer absorbance measurements on the cells within the 2D and 3D ICC scaffolds on days 2, 4, 6, 8, 12 and 14. Results are reported as mean ± SD (*n* = 3). **p < 0.01 compared to 2D cultures. (B) Live/dead staining of cells at days 2, 6 and 12. Live cells were stained with calcein-AM (green), and dead cells were stained with EthD-1 (red). Images were taken using a confocal microscope with a 10× lens. Scale bar is 50 μm. (C) 3D reconstructions using ZEN software of ICCs Live/Dead images taken at day 12 using a confocal microscope with a 20× lens. The orange dashed line indicates the perimeter of the pore cavities.

A quantitative analysis of cell proliferation with the CCK-8 assay (Fig. 3A) revealed cell number and cellular metabolic activity. The absorbance in 2D culture doubled from day 2 to day 4, while remained almost constant from day 6 to day 14, probably due to space restrictions for cell spreading, as cells had clearly reached confluence at day 8. Also, the absorbance in 2D culture drastically augmented compared to the Bare-ICC, Col-ICC and FN-ICC groups, most probably as a result of both increased cell metabolic activity and, differently from 3D cultures, increased cell viability. In addition, we noted a significant increase of absorbance in the FN-ICC group from day 4 to day 14 compared to the Bare-ICC and Col-ICC groups.

Cell viability was also analyzed by calcein AM/EthD-1 cell staining and confocal laser microscopy imaging (Fig. 3B and C). Live cells were stained with calcein (green fluorescence), while dead cells were stained with EthD-1 (red fluorescence). Throughout the experimental period (14 days), the majority of cells were living cells (green spots), whereas dead cells (red spots) were scarce. Even though CCK-8 data suggests the number of live cells in 2D culture increased over time, cells exhibited a fibroblastic morphology (elongated shape) at day 6. In 3D cultures, as the cells were not located on a single focal plane, it was not as easy to identify the cell shapes as in 2D culture, but the overall morphological changes were still visible. In particular, as already observed under the optical microscope 24 h after seeding, cells attached to the pore wall cavities in both functionalized ICCs and formed sheet-like structures over time, especially in the FN ICCs group. In the bare ICCs, instead, cells formed spheres at the center of the pores over time (Fig. 3C). When the cells from the 2D, Bare, Col and FN groups were compared, a significant increase in cell density in the FN group was observed. In the liver ECM, fibronectin forms a bridge between cells and collagen.^32^ This could provide an explanation for the reported enhanced spreading of hepatocytes on a fibronectin surface compared to a collagen surface.^45,46^

### 3.3 Cell functionality

**Fig. 4:**
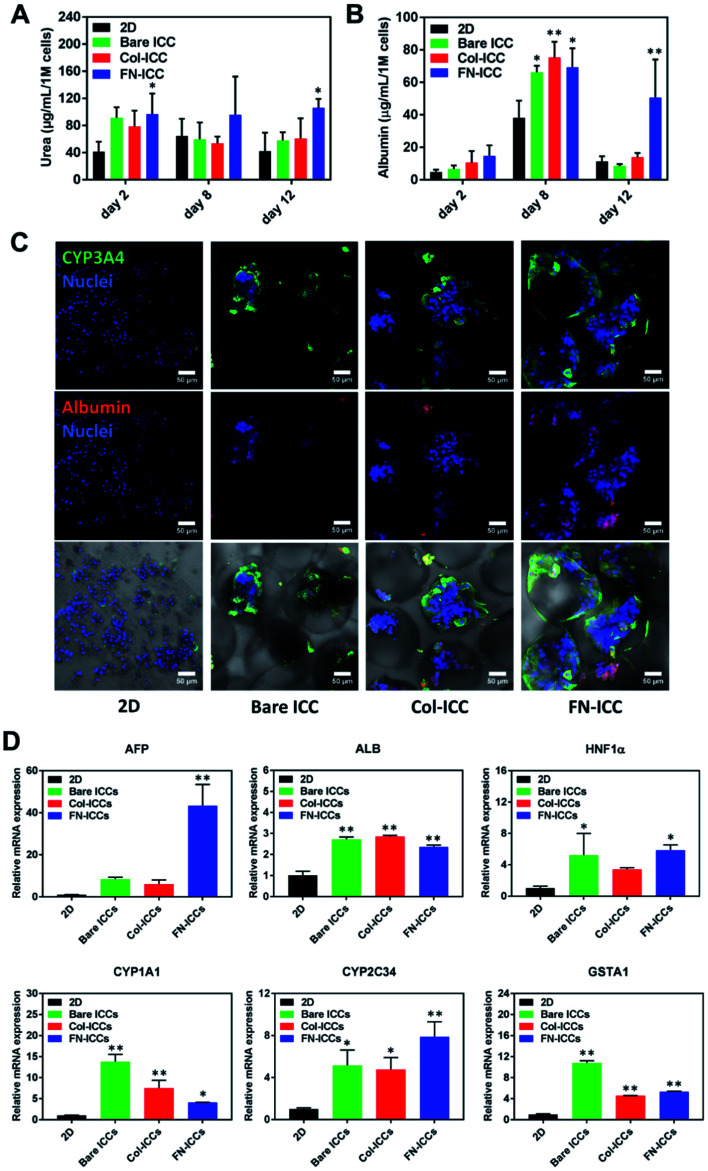
Evaluation of spatial liver-specific functions of pig hepatocytes. (A and B) Evaluation of pig hepatocytes albumin and urea secretion in 2D and 3D ICC scaffolds at day 2, 8 and 12. The results are mean ± SD (*n* = 3). *p < 0.05 and **p < 0.01 compared to 2D culture. (C) Immunofluorescence staining of CYP3A4 and albumin in cells on 2D, PEGDA scaffolds, Col I-coated scaffolds and fibronectin-coated scaffolds, on day 14. CYP3A4 was stained with Alexa Fluor 488 (green), albumin was stained with Alexa Fluor 594 (red), and cell nuclei were stained with H33342 (blue). Scale bar = 50 μm. (D) Gene expression of functional liver markers in hepatocytes on ICC scaffolds. Quantitative real-time PCR analysis of the mRNA of specific genes was conducted, and the data were normalized to the housekeeping gene GAPDH. Fold change comparison between the ICC groups and 2D culture; *p < 0.05 and **p < 0.01 compared to 2D culture.

To evaluate the liver-specific functions of pig hepatocytes, we tested the albumin and urea secretion at day 2, 8 and 12, and noted a higher albumin production and urea synthesis in the 3D ICC groups compared to the 2D group (Fig. 4A and B). In particular, the albumin production rate of cells cultured on a fibronectin-coated scaffold last longer and was much higher than that of cells on either a collagen-coated or a bare scaffold (Fig. 4B, day 12). This observation confirmed previous findings from a study of rat hepatocytes in monolayer culture.^47^ Similarly, we observed that the albumin expression level was stronger in the FN-ICC group compared with the Bare ICC and Col-ICC groups by using immunofluorescence staining under a confocal microscope (Fig. 4C).

In addition, the effect of 3D ICC culture on hepatocyte-specific gene expression was quantified by RT-qPCR after total RNA extraction. Compared with the 2D culture group, the mRNA levels for AFP, HNF1α, ALB, GSTA1, CYP1A1, and CYP2C34 were significantly up-regulated in all the ICC scaffolds (Fig. 4D). AFP and ALB are two markers of hepatocyte differentiation. Conversely, to what observed in the albumin secretion in the cell culture supernatant, the ALB gene expression levels in the FN-ICC hydrogels were not significantly higher than the other two ICC groups. Also, the hepatocytes marker HNF-1α, required for liver development and the expression of many liver-specific genes, was similarly upregulated in the 3D ICC groups.

Cytochrome P450 enzymes are critically associated with drug metabolism of the liver. In particular, CYP3A and 2C are responsible for metabolizing approximately 60% and 19% of the drugs in the clinic, respectively, and thus are regarded as the most important drug metabolizing enzymes in hepatocytes. CYP1A1 and CYP2C34 members of the cytochrome P450 subfamily involved in Phase I xenobiotic metabolism,^48^ and the GSTA1 involved in Phase II xenobiotic metabolism, displayed the higher mRNA expression in all the ICC groups (Fig. 4D). We also found that in 3D culture groups the expression of CYP3A4, another member of the cytochrome P450 subfamily involved in Phase I xenobiotic metabolism,^49^ examined by using immunofluorescence staining under a confocal microscope, was more visible than in the 2D group (Fig. 4C). No changes were instead detected for the regulatory gene HNF-4α, the phase I metabolic enzyme CYP3A29 and the phase II metabolic enzymes GSTA2 and GSTA4 (data not shown).

### 3.4 Drug toxicity studies

In order to evaluate the ability of the ICC platforms to detect the hepatotoxic effects of drugs, the viability and functionality of 2D and 3D cultured hepatocytes were assessed after single or repeated exposure to diclofenac. Numerous studies, both *in vitro* and *in vivo*, have shown that diclofenac undergoes aromatic hydroxylation and glucuronidation by phase I and II metabolic enzymes and that the formed reactive metabolites might be related to hepatotoxicity.^50^

**Fig. 5:**
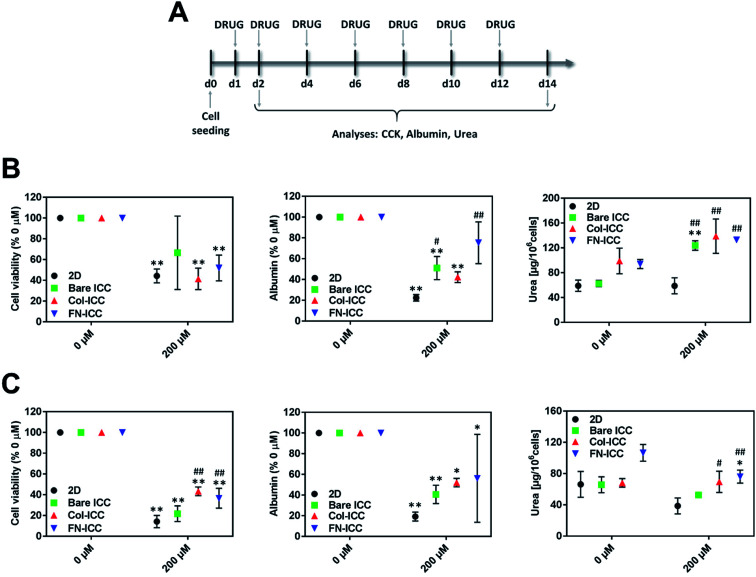
Long-term toxicity testing with ICC platforms. ICCs platforms detect diclofenac-induced toxicity. Hepatocytes in 2D and ICCs hydrogels were treated with 200 μM diclofenac for up to 14 days and acute and chronic toxicity effects were evaluated after 1 (24 h) or 7 (14 days) drug exposures. (A) Experimental timeline for the drug toxicity studies. (B) Cell viability, albumin, and urea production were assessed 24 h after diclofenac treatment. (C) Cell viability, albumin, and urea production were assessed after 7 repeated drug administrations. Data are reported as mean ± SD (*n* = 3). Cell viability and albumin data are normalized to the respective untreated groups. *p < 0.05 and **p < 0.01 compared to the respective 0 μM values; ^#^*p* < 0.05 and ^##^*p* < 0.01 compared to 2D 200 μM values.

Hepatocytes were allowed to recover from the seeding procedure for 24 h and then exposed to 200 μM diclofenac. Acute toxicity was assessed after 1 day, while chronic toxicity was assessed after 7 drug re-dosing over a period of 14 days (Fig. 5A) since we have shown that hepatocytes functionality in the ICC hydrogels could be maintained up to 2 weeks. Diclofenac cytotoxicity was evaluated by mean of cell viability, albumin secretion, and urea production.

In all the platforms tested and at each time point, hepatocyte viability decreased when the drug was added to the culture media. After treating the platforms with diclofenac for 24 h, cell viability was almost halved (Fig. 5B). Although no statistically significant differences were observed among the various treated systems after one single dose, cells in biofunctionalized ICCs showed reduced sensitivity than cells in 2D and bared PEGDA ICCs after repeated drug exposures. Also, drug re-dosing reduced further hepatocyte viability, with the biggest reduction observed in 2D and bare ICCs (Fig. 5C).

Similarly, after 1 single drug dose administration, albumin secretion in bare ICC and Col-ICC scaffolds was almost half than the respective non-treated cultures (Fig. 5B). Albumin production capability appeared least affected by drugs when hepatocytes were cultured in FN-ICCs and mostly affected when cells were cultured in conventional 2D multiwalls. However, irrespective of the number of doses administered, the capability of cells cultured in ICCs to produce albumin was higher than in 2D cultures. As in the case of cell viability, repeated drug administrations reduced further albumin secretion (Fig. 5C).

After 24 h of drug administration (Fig. 5B), urea secretion increased in the presence of diclofenac in all the ICC, while it remains almost unchanged in 2D. In particular, urea production was 2-fold higher in ICCs than in 2D when hepatocytes were exposed to diclofenac, and no significant statistical differences were observed among the treated ICCs. However, in all the platforms urea production was much lower when cells were exposed to multiple drug doses compared to a single dose. Compared to non-treated cells, drug re-dosing also led to a decrease in urea production in all the platforms except in the Col-ICCs (Fig. 5C), though urea production was still higher in ICCs than in 2D. These results are in agreement with previous studies performed by De Bartolo and colleagues, who reported that when porcine hepatocytes seeded onto polyethersulfone membranes were repeatedly exposed to daily doses of diclofenac, urea synthesis initially increased, reaching a maximum after four days of culture, and then gradually decreased.^51^

Both after acute and chronic drug exposure, hepatocytes cultured on ICC scaffolds showed reduced sensitivity to drug toxicity compared to 2D monolayer culture, probably due to the specific hepatocyte arrangement in the ICC hydrogels, characterized by a well-developed ECM and densely packed cells with multi-planar cell–cell and cell–ECM interactions. Indeed, in the non-fouling bare PEGDA ICCs cells aggregated together at the center of the scaffold cavities and over time formed hepatospheres while in the biofunctionalized PEGDA ICCs cells adhered to the hydrogel pore walls and over time aggregated and proliferated to form multi-sheet like cell aggregates. Such a pattern was also observed in previous studies which reported that HepG2 cells cultured in 2D conventional tissue culture plates showed increased sensitivity to diclofenac toxic effects compared to HepG2 spheroids.^52^ The different cell arrangement within the various ICC hydrogels might also explain the diverse sensitivity to drug toxicity observed between bare and biofunctionalized ICC scaffolds after chronic drug exposure, but not after acute drug exposure. After 48 h of culture, cells in all the ICC are almost single cells starting to aggregate and form clusters, as visible in Fig. 3B, so that cells and ECM shielding effects are negligible and almost no significant differences in cell viability are visible among all the cultures. However, already after six days of culture, the different cell arrangements are obvious (Fig. 3B and 4C), potentially resulting in diverse drug sensitivity. Besides the differences in cell architecture, the differences in liver-specific cell functionality observed between 2D and 3D cultures might also contribute to explain the diverse toxic effect exerted by diclofenac in the two types of cultures. Our results indicate that phase I and II metabolic enzymes are expressed to a greater extent in ICCs than in 2D cultures and thus that hepatocytes cultured onto 3D hydrogels might possess an increased ability to metabolize diclofenac. Although many studies have linked diclofenac toxicity to its metabolites, studies have also shown that high doses of the drug saturate the cytochrome P450 enzyme system^51,53^ and that not only the metabolites but also the drug itself might affect cell viability.^54,55^ In particular, studies have shown that the drug itself might compromise mitochondrial ATP synthesis,^54,55^ probably as a result of the uncoupling effect on the mitochondrial oxidative phosphorylation, due to the drug protonophoretic activity,^56^ and the subsequent drug-induced mitochondrial membrane potential decrease.^50^ These mechanisms might contribute to explain the different toxic effects observed in 2D and 3D cultures and also the much lower albumin production observed in 2D cultures, as substantial amounts of ATP are required for albumin synthesis. Therefore, diclofenac metabolites might be more involved in the hepatotoxicity observed in the 3D cultures.

## 4. Conclusions

In this study, using 3D ICC scaffolds, we evaluated the porcine hepatocytes viability, morphology, hepatic-specific functions, and patterns of gene expression; and established a functional porcine hepatocyte culture platform that can be used for predicting drug toxicity. In comparison to 2D cell platforms, three-dimensional (3D) ICC scaffolds culture environment and the presence of extracellular matrix (ECM) proteins (collagen type I or fibronectin), especially the fibronectin, facilitate hepatocyte viability and maintenance of the liver-specific phenotype *in vitro*, and enable prediction of hepatotoxicity caused by diclofenac. This work shows the potential of the ICC scaffold, as they could have significant implications for the design of *in vitro* liver models for applications such as tissue regeneration, toxicity studies, and liver disease therapy.

## Conflicts of interest

The authors declare no conflict of interest.

## Supplementary Material
